# Symptomatic adjacent segment disease after single-lever anterior cervical discectomy and fusion: Incidence and risk factors

**DOI:** 10.1097/MD.0000000000008663

**Published:** 2017-11-27

**Authors:** Feng Wang, Hong-Tao Hou, Peng Wang, Jing-Tao Zhang, Yong Shen

**Affiliations:** a Department of Spine Surgery, The Third Hospital of Hebei Medical University, The Key Laboratory of Orthopedic Biomechanics of Hebei Province; b Department of Gastroenterology, Hebei General Hospital, Shijiazhuang, China.

**Keywords:** adjacent segment degeneration, adjacent segment disease, anterior cervical discectomy and fusion, developmental canal stenosis, risk factors

## Abstract

The purpose of this study was to determine the incidence and risk factors of symptomatic adjacent segment disease (ASD) following single-lever anterior cervical discectomy and fusion (ACDF) for cervical degenerative diseases.

From January 2000 to December 2010, a total of 582 patients with cervical radiculopathy and myelopathy who had undergone single-lever ACDF surgery in the authors’ institution were reviewed retrospectively. Patients who had a revision surgery for symptomatic ASD were selected for this study. The authors analyzed the incidence for ASD after single-lever ACDF. And univariate analysis and logistic regression analysis were performed to identify the risk factors of ASD.

Among the 582 patients, 36 patients received subsequent surgical management for ASD after initial single-lever ACDF for an overall prevalence of 6.2%. The average onset time of ASD was 8.5 (2–15) years. The univariate analysis showed that there were no significant differences in sex, duration of disease, BMI, DM, smoking, operative levels, and follow-up period (*P* > .05) between the 2 groups with and without ASD. There were statistically significant differences in age at the time of operation (χ^2^ = 4.361, *P* = .037), and developmental canal stenosis (χ^2^ = 4.181, *P* = .041) between patients with and without ASD. The variables of age at the time of operation and developmental canal stenosis were included in a logistic regression model. The logistic regression analysis revealed that age at the time of operation ≤50 years (*P* = .045, OR = 3.015, 95% CI *=* 1.024–8.882) and developmental canal stenosis (*P* = .042, OR = 2.797, 95% CI *=* 1.039–7.527) were the risk factors for ASD after single-lever ACDF.

In the present study, the incidence of symptomatic ASD after single-lever ACDF was 6.2%. And the age at the time of operation ≤50 years and developmental canal stenosis were the risk factors for ASD. The patients ≤50 years old at the time of operation or with developmental canal stenosis are more likely to develop ASD after surgery, and the risk of reoperation will increase.

## 1 Introduction

Anterior cervical discectomy and fusion (ACDF), which was first described in 1950s, is widely accepted as a standard surgical treatment for cervical spondylosis refractory to conservative management.^[1,2]^ ACDF allows direct decompression of the neural elements and generally is accompanied by interbody fusion and anterior plate stabilization. However, clinical and biomechanical studies suggested that adjacent level kinematic might predispose to adjacent segment degeneration after ACDF.^[3]^ In several studies, the adjacent segment degeneration rates varied from 25% to 92% during a long follow-up period.^[4–6]^ Adjacent segment disease (ASD), which is clinical and symptomatic adjacent segment degeneration, has been recognized as an important entity after ACDF.^[7–9]^ Lawrence et al^[10]^ estimated the rate of ASD in the cervical spine after ACDF to be between 1.6% and 4.2% per year. Recently, Lee et al^[11]^ reported adjacent segments underwent surgical treatment at an annual rate of 2% after cervical fusion and predicted that 22% of patients would need a reoperation for ASD within 10 years. Therefore, the incidence of ASD is significantly lower than that of adjacent segment degeneration. Previous studies have demonstrated that the development of adjacent segment degeneration may be influenced by several factors, including the age, smoking history, number and location of fusion segments, plate-to-disc distances, excessive disc space distraction, kyphotic malalignment, and so on.^[11–14]^ However, to the best of our knowledge, there have been controversies about the exact incidence of ASD after ACDF and its risk factors. In this study, the patients underwent a revision surgery for ASD after single-lever ACDF were retrospectively analyzed. The authors described the incidence of ASD after single-lever ACDF based on the past 10-year experience, and tried to investigate the risk factors associated with ASD according to the preoperative data.

## 2 Materials and methods

### 2.1 Study population selection

**Figure 1:**
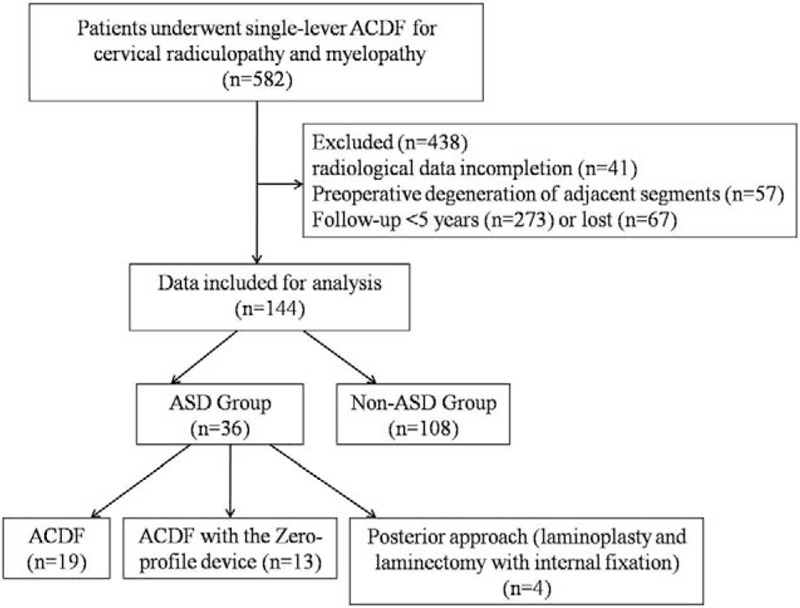
Flow diagram showing patients allocation in the present study. ACDF = anterior cervical discectomy and fusion, ASD = adjacent segment disease.

From January 2000 to December 2010, a total of 582 patients underwent single-lever ACDF for cervical radiculopathy and myelopathy in the authors’ institution. We retrospectively reviewed the patients who had undergone single-lever ACDF for cervical degenerative diseases between C3 and C7 and had at least 5 years of follow-up. Finally, 144 patients were included in this study. Among them, 36 patients were identified who had a revision surgery for ASD between January 2011 and December 2015 (Fig. 1). The patients’ age at the time of single-lever ACDF ranged from 31 to 74 years (average 48.3 years), duration of disease ranged from 6 to 54 months (average 35.6 months), and the follow-up periods ranged from 5 to 15 years (average 7.4 years). All the patients had no expression of already existing degeneration (radicular or myelopathic signs and symptoms that correlate with imaging evidence of degeneration) at the time of the first surgery. Furthermore, these patients who developed gradual neurological changes followed 6 months of invalid conservative treatment. However, the patients with cervical spine trauma, tumor spinal pathologies, neoplasm, spinal infections, congenital deformations, and chronic systemic illnesses such as rheumatoid arthritis and neurodegenerative diseases were excluded from this study. This study had been approved by Ethics Committee of The Third Hospital of Hebei Medical University.

### 2.2 Surgical management

Under general anesthesia, standard ACDF was performed on each patient by the same senior surgeon. After complete discectomy and osteophytectomy were carried out, the endplate cartilage was symmetrically removed with a high-speed drill and curette until bleeding occurred. In all cases, an adequate decompression of cervical cord and the origin of nerve root were obtained. After confirming good pulsation of the thecal sac, a cervical titanium cage filled with autologous bone grains or autograft of bone was inserted into the intervertebral space to obtain firm interfusion. And the anterior plate system was applied to provide stability until bony fusion. Ambulation was allowed on the second day after surgery, whereas external immobilization of the cervical spine was kept for 2 months with a cervical collar.

A revision surgery should be considered for the patients with obvious clinical manifestation and poor conservative treatment of ASD after single-lever ACDF. In all, 36 patients received reoperation by the same senior surgeon. According to the clinical situation, initial operation and secondary preoperative imaging findings were analyzed comprehensively, the surgical approaches were used by ACDF, ACDF with the Zero-profile device, laminoplasty and laminectomy with internal fixation.^[15]^

### 2.3 Evaluation criteria

Clinical data including clinical and radiological evaluation results were collected preoperatively at 3, 6, 12, and final follow-up after single-lever ACDF. All patients were followed up for at least 5 years after first surgery. All data regarding age, sex, duration of disease, body mass index (BMI), diabetes mellitus (DM), smoking, operative levels, follow-up period, developmental canal stenosis, and the way bone graft were reviewed and statistically analyzed. The sagittal canal diameter was measured on lateral neutral radiographs from the middle portion of the posterior surface of the vertebral body to the innermost cortical surface of the lamina. The anteroposterior diameter of the cervical canal was defined as the average of sagittal canal diameters from C3 to C7. Male patients whose sagittal diameter was <14 mm and that of female patients was <13 mm at least at one level indicated the existence of developmental canal stenosis.^[16]^ Serious adverse events were those that could influence clinical result, such as loosening of the implant, collapse of the fusion intervertebral space, hematoma, and deep infection. Successful fusion was defined as R4 degrees of angular motion on flexion and extension radiographs, the presence of bridging trabecular bone between the fused vertebrae, and the absence of any radiolucent zones spanning <50% of the implant-vertebral interface on CT images. Two independent radiologists assessed the radiographs. In the event of disagreement about fusion healing, a third independent reading was obtained.

### 2.4 Statistical methods

All data were collected, and the software of by SPSS Version 17.0 (SPSS Inc, Chicago, IL) was used for the statistical evaluation. Results were presented as mean ± SD. Univariate analysis were performed to examine the relationship between outcome at the final follow-up and prognostic factors. Chi-square tests were used for nominal variables and Mann-Whitney tests were used for continuous variables. Variables were included in a logistic regression model if their univariate analysis *P* value was <.05. The threshold for significance was a *P* value of <.05.

## 3 Results

Among the 582 patients, 36 patients received subsequent surgical management for ASD after initial single-lever ACDF for an overall prevalence of 6.2%. The average onset time of ASD was 8.5 (2–15) years. Of those, 23 patients had a single adjacent segment level, 10 patients had 2 levels, and 3 patients had 3 levels. The adjacent levels were located at C3–4 in 8 patients, C4–5 in 15 patients, C5–6 in 14 patients, and C6–7 in 16 patients. ASD occurred superior to the prior fusion in 18 patients, inferior in 13 patients, and at both adjacent levels in 5 patients (Table 1). Among them, 19 patients underwent ACDF surgery, 13 patients underwent ACDF with the Zero-profile device, and the other 4 patients underwent laminoplasty or laminectomy with internal fixation. All patients were effectively relieved of spinal cord compression and improved spinal cord function after the revision surgery.

**Table 1:**
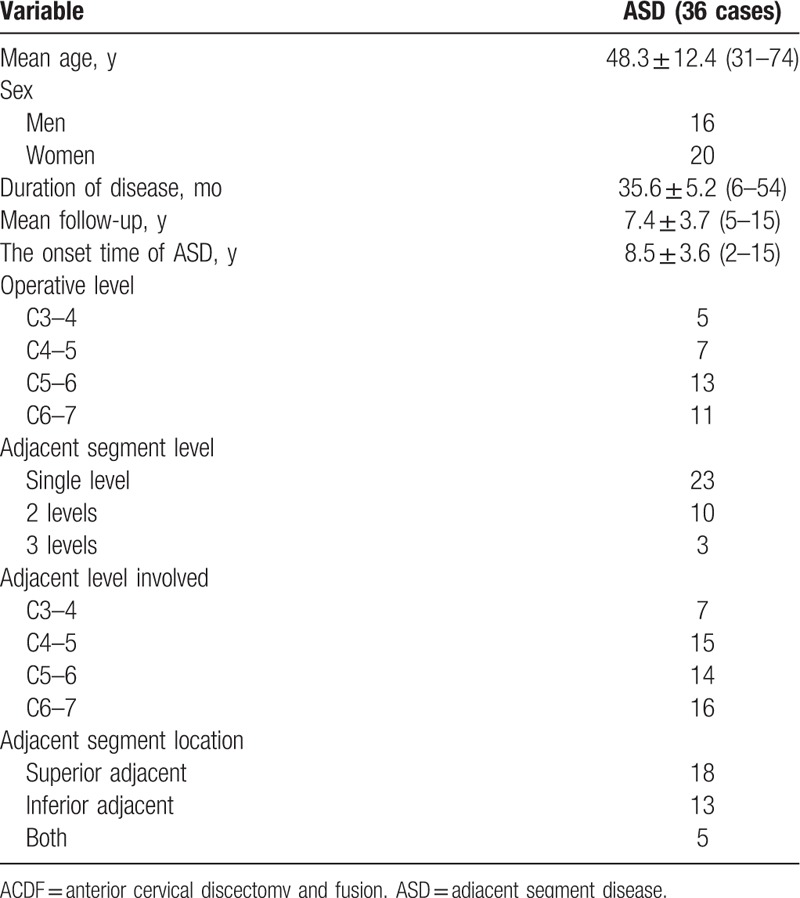
Demographic data of patients with ASD after single-lever ACDF.

The univariate analysis showed that there were no significant differences in sex, duration of disease, BMI, DM, smoking, operative levels, and follow-up period (*P* > .05) between the 2 groups with and without ASD (Table 2). There were statistically significant differences in age at the time of operation (*χ*^*2*^ = 4.361, *P* = .037), and developmental canal stenosis (χ^2^ = 4.181, *P* = .041) between patients with and without ASD (Table 2). The variables of age at the time of operation and developmental canal stenosis were included in a logistic regression model. The logistic regression analysis revealed that age at the time of operation ≤50 years (*P* = .045, OR = 3.015, 95% CI *=* 1.024–8.882) and developmental canal stenosis (*P* = .042, OR = 2.797, 95% CI *=* 1.039–7.527) were the risk factors for ASD after single-lever ACDF (Table 3).

**Table 2:**
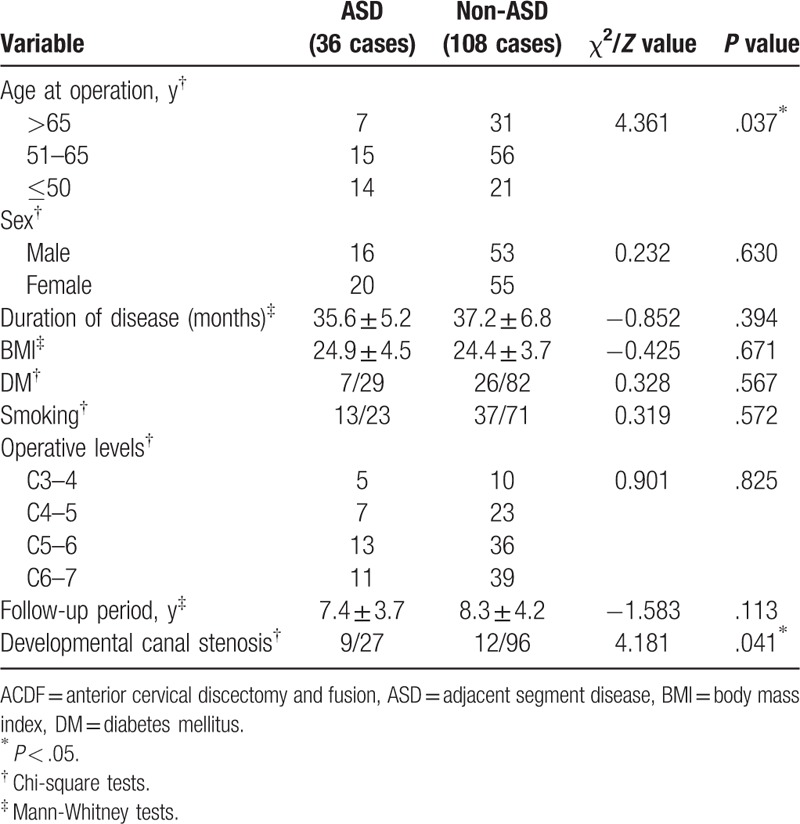
Comparison between the patients with and without ASD after single-lever ACDF.

**Table 3:**
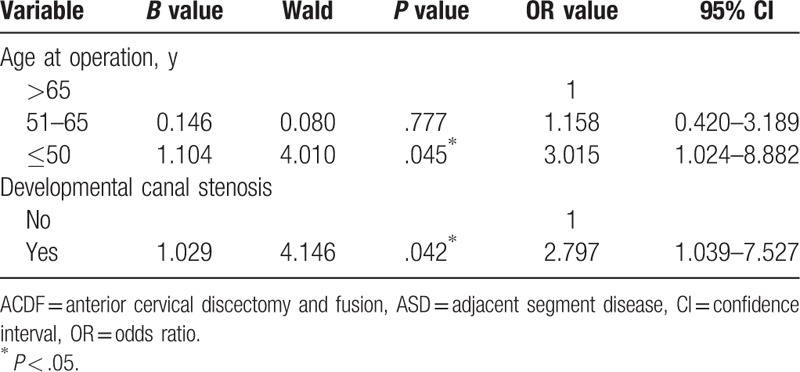
Logistic regression analysis for symptomatic ASD after single-lever ACDF.

## 4 Discussion

Adjacent segment degeneration is the radiological degenerative change that occurs adjacent to the fusion lever, and ASD is the condition which presents with myelopathic or radiculopathic symptoms caused by such degenerative changes.^[17,18]^ In the past few decades, an increasing number of studies and data show that ASD after ACDF had become a considerable challenge for surgeons. A review of the literatures, the rate of reoperation of ASD patients undergoing ACDF for cervical radiculopathy and myelopathy ranged from 2.1% to 22%.^[9,11,15,19]^ These results are largely in line with the study by Hilibrand^[5,9]^ which monitored the ASD development after ACDF and found an incidence of approximately 3% per year. Otherwise, Hilibrand^[5]^ also predicted that 25.6% of the patients will develop ASD after ACDF, of which two-thirds required a revision surgery. In this long-term study, 6.2% (36/582) of the patients were involved to undergo reoperation for ASD after single-lever ACDF. The reoperation rate was similarly compared with previous studies. And the average onset time of ASD was 8.5 years. The incidence of ASD is significantly lower than that of adjacent segment degeneration. Therefore, not all adjacent segment degeneration patients are required to undergo a reoperation treatment. However, during the follow-up, we have excluded the patients with radiological data incompletion, preoperative degeneration of adjacent segments, and the follow-up of <5 years or lost. Therefore, the actual incidence of ASD after ACDF in this study will be higher.

Previous studies have demonstrated that the development of adjacent segment degeneration may be influenced by several factors, including the age, smoking history, number and location of fusion segments, plate-to-disc distances, excessive disc space distraction, kyphotic malalignment, and so on.^[11–14]^ Nonetheless, there have also been controversies about the risk factors of ASD after ACDF. It remains unclear whether ASD is caused by the natural progression of aging or the biomechanical impact of the interbody fusion. According to the preoperative data, we tried to investigate the risk factors associated with ASD after single-lever ACDF. In the present study, the univariate analysis showed that there were no relationships between the 2 groups with and without ASD in sex, duration of disease, BMI, DM, smoking, operative levels, and follow-up period. However, the age at the time of operation and developmental canal stenosis were closely related to the occurrence of ASD. Moreover, the logistic regression analysis revealed that age at the time of operation ≤50 years and developmental canal stenosis were the risk factors for ASD after single-lever ACDF.

At present, there is controversy about whether the age is a risk factor for ASD after ACDF. Olsewski^[20]^ showed that the older patients were more likely to develop ASD after surgery, and the risk of reoperation is increased. Ahn et al^[21]^ observed 64 patients who underwent single-level ACDF with a follow-up time of 3 years and reported that the patients over the age of 50 were at higher risk of developing ASD after ACDF. However, Jawahar^[22]^ and van Eck^[23]^ found that the age did not affect the development of ASD after ACDF. This correlates with the findings from Hilibrand et al^[5]^ who analyzed that the age was not a risk factor for ASD. Despite the physiological aging of the cervical spine, several cross-sectional and longitudinal studies have been observed in healthy volunteers.^[7,8]^ And Kellgren et al^[24]^ suggested that by the time a normal population was 50 years old, the radiographs will show degenerative changes of the cervical spine in approximately 50%. Different with previous studies, in the present study, the logistic regression analysis revealed that the age at the time of operation ≤50 years (OR = 3.015) was a risk factors for ASD after single-lever ACDF. The result of this study showed that the younger patients treated by ACDF surgery, the more easily they were to undergo an ASD that required a reoperation treatment. We believed that this is because the fusion of adjacent segments accelerates the natural process of degradation and leads to the emergence of ASD after single-lever ACDF. Moreover, we presumed that higher risk in the younger population may reflect greater physical demands or higher expectations of physical function on their part. Conversely, the older age is more likely to have other medical comorbidities precluding them from having a reoperation. Therefore, with the development of minimally invasive technique, for younger patients (≤50 years), such as posterior key-hole surgery and artificial disc replacement, may reduce the incidence of ASD and reoperation compared with ACDF.

A considerable amount of literature had been published on the issue that the developmental canal stenosis may be an important factor of adjacent segment degeneration after ACDF. Zhang et al^[25]^ reported that developmental canal stenosis can increase the rate of radiographic adjacent segment degeneration after initial ACDF and has the highest validity for predicting radiographic adjacent segment degeneration. Furthermore, Eubanks et al^[26]^ found that although developmental canal stenosis increases the incidence of radiographic adjacent segment degeneration, it does not appear to predict symptomatic ASD. However, our study indicated that developmental canal stenosis is also a risk factor for ASD after single-lever ACDF, with an OR value of 2.797. A narrowed spinal canal may cause new compression of the spinal cord and lead to the development of myelopathy after initial ACDF. Therefore, the risk of ASD should be evaluated before performing ACDF in patients with developmental cervical canal stenosis.

## 5 Conclusion

In our study, the incidence of symptomatic ASD after single-lever ACDF was 6.2%. However, the actual incidence of ASD after ACDF in this study will certainly be higher. In addition, the age at the time of operation ≤50 years and developmental canal stenosis were the risk factors for ASD. The patients of younger age at the time of operation or with developmental canal stenosis are more likely to develop ASD after surgery, and the risk of reoperation will increase. We suggested that patients ≤50 years of age at the time of operation or with developmental canal stenosis should be told of the risks of ASD before ACDF surgery. However, this study was only a retrospective study with a small sample size to explore the risk factors for ASD after single-lever ACDF. There may be a selection bias resulting in this finding. And there is still a need for a large sample multicenter study to further confirm this result.
